# Public Safety and Faulty Flood Statistics

**DOI:** 10.1289/ehp.12042

**Published:** 2008-12

**Authors:** Robert E. Criss, William E. Winston

**Affiliations:** Department of Earth and Planetary Sciences, Washington University, St. Louis, Missouri, E-mail: criss@wustl.edu

We thank Black (2008) for his thoughtful assembly of diverse perspectives including ours on why another disastrous Mississippi River flood occurred so soon after 1993. We have an update that elucidates and quantifies several key points of his article, particularly that *a*) flood frequency and heights are increasing, *b*) water levels for regulatory “100-year” floods are profoundly underestimated, and *c*) misconceptions about risk confound appropriate responses to flooding.

At many sites in Iowa and Missouri the Flood of 2008 neared or exceeded the record “200-year” or “500-year” levels attained in 1993 [National Weather Service (NWS) 2008; U.S. Army Corps of Engineers (USACE) 2008b], prompting many to wonder how two such events could occur in only 15 years. Defenders of flow-frequency predictions cite their rigorous methods and assure us that improbable outcomes are possible, even two “100-year” floods in consecutive years (U.S. Geological Survey 2005). Such arguments miss the key issue: Are the flood probabilities calculated by USACE (2004, 2008a) credible?

Problems with the USACE (2004, 2008a) probabilities are exemplified by recent flooding at Hannibal, Missouri (Figure 1). The record stage set in 1993 exceeded the calculated 500-year level, whereas 2008 was a 200-year event. In addition, 2001 suffered a 50- to 100-year flood, 1986 and 1996 experienced 25- to 50-year floods, and five more years had 10-to 25-year floods. Are these calculated recurrence intervals reasonable, or is it more likely that the dice, in effect, are loaded?

Statistically, two 200-year floods would likely not occur in an interval < 330 years, but Hannibal has recently had far worse (Figure 1). Two 500-year floods would probably not occur in < 840 years, yet two such floods recently occurred at Canton, Missouri, and Burlington, Iowa. A chi-square statistical test rejects the assumed correctness of the USACE frequencies with 99.9% confidence. The 100-year flood stage at Hannibal should be realistically redefined as a 10-year flood, as reported by Black (2008).

Because floods are becoming more frequent and more severe over much of the Mississippi River basin (Black 2008; Criss and Shock 2001; Remo and Pinter 2007), statistical calculations based on the historical record are not appropriate predictors of future flooding, particularly for extreme events (Klemes 2000). The likelihood of attaining a given flood stage today is not the same as it was a century ago, and besides, the record is far too short to calculate what a 200-year or a 500-year flood might be.

As Black (2008) discussed, calculated flood probabilities are not merely academic. The Federal Emergency Management Agency (FEMA) and the National Flood Insurance Program use these calculations to delimit 100-year flood zones and to set insurance requirements and rates. Understated risk burdens everyone with debt (Crichton 2002; Davidson 2005) and places humans and property at risk from water that is not only too high but also laden with contaminants and sediment. Unfortunately, erroneous calculations (USACE 2004, 2008a) are being used in the very latest proposals (USACE 2008c) for flood protection and ecosystem restoration. We need to use realistic concepts about flooding in our management plans.

## Figures and Tables

**Figure 1:**
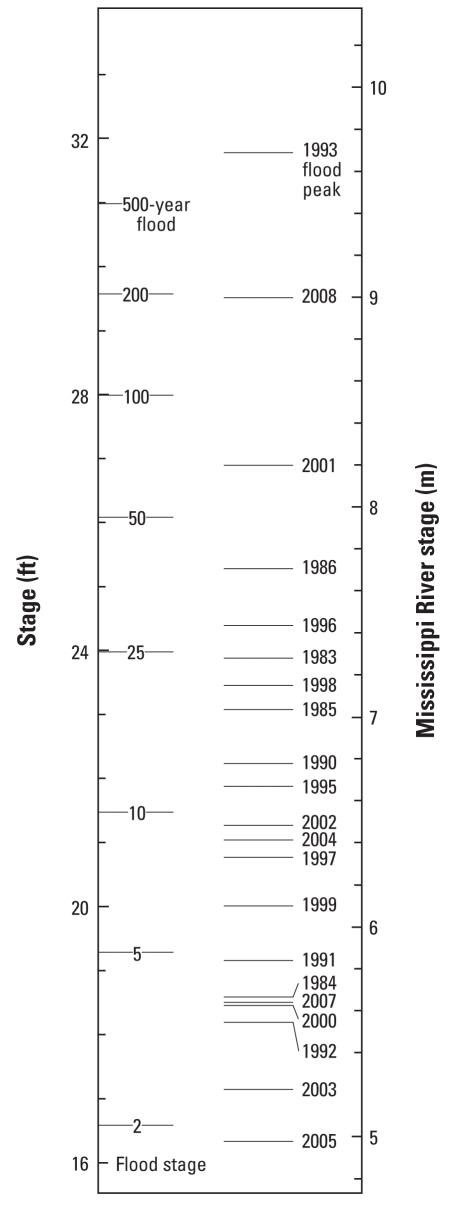
Mississippi River flood levels recorded at Hannibal over the last 25 years [right (NWS 2008; USACE 2008b)] compared with the theoretical stages for 2-year to 500-year floods [left (USACE 2004, 2008a)].
